# Calcium-alkali syndrome as a rare cause of severe hypercalcemia requiring dialysis in early twin gestation

**DOI:** 10.1177/1753495X221145574

**Published:** 2022-12-18

**Authors:** P Beamish, C Mansour, I Druce, P O’Meara

**Affiliations:** 1 Department of Medicine, Division of Endocrinology and Metabolism, 6363 University of Ottawa, Ottawa, Canada; 2 Department of Medicine, Division of General Internal Medicine, 6363 University of Ottawa, Ottawa, Canada

**Keywords:** Hypercalcemia, calcium-alkali, pregnancy, twin gestation

## Abstract

Hypercalcemia is rare in women of child-bearing age, and most cases are due to primary hyperparathyroidism. A 28-year-old woman, 14 weeks pregnant with dichorionic diamniotic twins, presented to hospital with vomiting, muscle cramps, and weakness. She had been taking calcium carbonate for gastric reflux and nausea from 5 weeks of gestation. Investigations revealed severe hypercalcemia, metabolic alkalosis, and renal injury. She was transferred to intensive care, receiving fluid resuscitation and subcutaneous calcitonin followed by dialysis. Investigations revealed suppressed PTH and PTH-related peptide, negative malignancy screening and low vitamin D level. Calcium and renal function quickly normalized and with cessation of calcium carbonate remained normal throughout the rest of pregnancy. Reports of calcium-alkali syndrome causing severe hypercalcemia are scarce, with most cases occurring later in gestation. This case represents a dramatic presentation requiring renal replacement therapy early in twin gestation.

## Introduction

Hypercalcemia presents rarely in pregnancy. One study of 300,000 women of childbearing age found that the incidence of moderate to severe hypercalcemia was 0.04%.^1^

Despite adaptations to calcium metabolism in pregnancy, the most common causes of hypercalcemia in pregnancy mirror those in the general population, with primary hyperparathyroidism accounting for over 90% of cases.^2^ Most other causes of hypercalcemia have been described in pregnancy in case reports.^1^ Symptoms of hypercalcemia in pregnancy include polydipsia, vomiting, constipation, and depressive symptoms, and can be misattributed to pregnancy itself. Hypercalcemia in pregnancy increases the risk of intrauterine growth restriction, fetal loss, neonatal death, neonatal hypocalcemia with tetany, and permanent hypoparathyroidism in the infant.^3^

Described here is a case of calcium-alkali syndrome, causing severe hypercalcemia in pregnancy.

## Case

A 28-year-old woman was pregnant for the first time, and had been found to have a dichorionic diamniotic twin pregnancy. She presented at 13 weeks’ gestation to the emergency department of a community hospital with several weeks of intractable vomiting (Day 0). Whilst blood tests were performed, the abnormal renal function, increased bicarbonate and hypochloraemia were not appreciated (Table 1). She was diagnosed with nausea and vomiting of pregnancy, received 1 L of 0.9% sodium chloride and was discharged with a prescription for oral metoclopramide. She returned to the same hospital six days later with worsening nausea and vomiting, general malaise, and muscle cramping.

**Table 1.** Laboratory investigations on presentations to hospital.

Variable	Units	Reference ranges		Presentations to hospital	
		Average adult	Second trimester^4^	First (Day 0)	Second (Day 6)
Venous blood gas					
pH		7.33–7.46	N/A	—	7.52
pCO2	mmHg	40–50	N/A	—	44
Bicarbonate	nmol/L	22–27	18–26	—	36
Base excess	mmol/L	−2.5–2.5	N/A	—	11.9
Ionized calcium	mmol/L	1.14–1.28	1.1–1.25	—	2.45
Hematology					
White blood cells	× 10^9^/L	3.5–10.5	5.6–14.8	19.4	20.2
Neutrophils	× 10^9^/L	2.0–7.5	3.8–12.3	16.1	16.6
Hemoglobin	g/L	115–155	97–148	108	97
Mean corpuscular volume	fL	80.0–100.0	85.8–99.4	86.0	85.3
Platelets	× 10^9^/L	130–380	155–409	358	387
Biochemistry					
Sodium	mmol/L	136–145	129–148	132	132
Potassium	mmol/L	3.5–5.1	3.3–5.0	4.0	3.9
Chloride	mmol/L	98–107	97–109	89	88
Bicarbonate	mmol/L	19–30	18–26	29	34
Anion gap	mmol/L	8–16	10–18	14	10
Plasma glucose	mmol/L	4.0–11.0	4.0–11.0	5.5	5.8
Urea	mmol/L	2.4–6.4	1.1–4.6	13.9	16.7
Creatinine	µmol/L	49–84	35–71	374	505
Phosphate	mmol/L	0.83–1.40	0.81–1.49	—	1.54
Calcium	mmol/L	2.24–2.58	2.05–2.25	—	5.03
Albumin	g/L	36–47	26–45	—	40
Magnesium	mmol/L	0.66–1.07	0.63–0.92	—	0.91
AST	U/L	9–25	3–33	28	42
ALT	U/L	6–30	2–33	48	60
ALP	U/L	46–118	25–126	—	103
Bilirubin	umol/L	≤ 11	1.7–13.7	—	7

The patient's past medical history was significant for gastroesophageal reflux disease (GERD), resolved reflux nephropathy, and childhood asthma. Her oral medications included folic acid 1 mg daily, calcium carbonate 750 mg, as needed, up to six times daily (300 mg of elemental calcium each), doxylamine-pyridoxine 10 mg-10 mg six tablets per day, and metoclopramide 10 mg two times per day. She discontinued her longstanding pantoprazole once pregnant due to safety concerns. Dietary calcium intake was modest, consisting of one cup of almond milk daily.

On re-presentation (Day 6), the patient had a temperature of 36.7 °C, blood pressure of 127/82 mmHg, respiratory rate of 20 breaths per minute, oxygen saturation of 97% on room air, and heart rate of 120 bpm. Routine blood tests revealed severe hypercalcemia, hyperphosphatemia, metabolic alkalosis, and renal injury (Table 1).

The patient was transferred to intensive care at a tertiary hospital. She was fluid resuscitated with 0.9% sodium chloride and given 400 units of subcutaneous calcitonin to acutely reduce calcium levels while avoiding fetotoxicity of bisphosphonates. She received a single run of sustained low efficiency dialysis the following day. Obstetric ultrasound confirmed live twin-gestation. When accounting for reference range changes in pregnancy, investigation into the etiology of hypercalcemia revealed suppressed parathyroid hormone (PTH), normal 25-OH vitamin D, and elevated thyroid hormones (Table 2). Parathyroid hormone-related peptide (PTHrP) was sent to assess for paraneoplastic or placental causes of hypercalcemia. In the meantime, a partial malignancy screen was undertaken and revealed unremarkable serum protein electrophoresis, computed tomography of the chest, magnetic resonance imaging of the abdomen and pelvis, and bilateral breast ultrasounds. Thyroid ultrasound revealed a 16 mm TIRADS 4 nodule, which proved benign on subsequent biopsy.

**Table 2.** Investigations into hypercalcemia etiology.

Parameter	Units	Reference intervals		Result
		Average adult	Second trimester^4^	
Thyroid stimulating hormone	mIU/L	0.27–4.20	0.37–3.60	0.08
Free T4	pmol/L	12.0–22.0	7.7–12.9	23.0
Free T3	pmol/L	2.8–6.8	6.2–6.5	7.1
Parathyroid hormone	pmol/L	1.6–6.9	1.9–2.6	1.2
Parathyroid hormone-related peptide	pmol/L	≤ 4.2	1.8–2.2	1.6
25-OH Vitamin D	nmol/L	75–250	25–55	68
1,25-OH Vitamin D	pmol/L	48–190	187–416	<12
β-human chorionic gonadotropin	IU/L			351,975

Calcium and renal function normalized by Day 8 and remained stable until discharge on Day 12. The patient was advised to stop taking calcium carbonate and calcium-containing supplements. Proton-pump inhibitor (PPI) therapy was resumed. PTHrP and 1,25-OH vitamin D results were low (Table 2), supporting the suspicion that PTH was not completely suppressed likely due to lab inaccuracy at the lower limit of detection. A diagnosis of calcium-alkali syndrome was confirmed.

The patient remained normocalcemic for the rest of her pregnancy and no further assessment or change in management was required. She delivered two healthy baby girls (APGAR 9 at 1 & 5 min for both, weight 2256 and 2922 g) by planned caesarean section at 37 weeks and 6 days of gestation.

## Discussion

There are physiologic changes in pregnancy which may go unrecognized if non-pregnancy reference ranges are used. During pregnancy, there is increased urinary loss of calcium from increased glomerular filtration, and syphoning of calcium and vitamin D for fetal bone mineralization.^5^ Despite increased calcium demand, and a fall in serum albumin and total calcium, ionized calcium (calcium unbound to albumin) concentrations remain unchanged due to multiple compensatory mechanisms including increased gastrointestinal absorption and bone resorption.^6^

Calcium-alkali syndrome is characterized by the triad of hypercalcemia, renal failure, and metabolic alkalosis caused by the co-ingestion of calcium and absorbable alkali.^6^ A 2011 summary of nine case reports of calcium-alkali syndrome in pregnancy revealed women typically in their third trimester, with severe hypercalcemia, and typically modest reversible renal injury.^7^ Seven of the cases had favorable outcomes, one fetus was stillborn at 37 weeks, and the last recovered from neonatal hypercalcemia, jaundice, and sepsis. Another case report documented calcium-alkali syndrome presenting only with reduced fetal heart rate variability on non-stress-test and resolution with conservative management.^8^

In comparison, the patient described here presented earliest, had the most severe degree of kidney injury, and had a twin gestation. This case illustrates the importance of recognizing and investigating acute kidney injury in pregnancy, interpreting lab results with pregnancy-specific reference ranges, as well as considering a broad differential diagnosis for the pregnant patient presenting with intractable vomiting. This patient's symptoms peaked at 13 weeks’ gestation, whereas hyperemesis gravidarum tends to peak around 9 weeks’ gestation.^9^ Twin gestations are associated with 1.5–2.5 times increased human chorionic gonadotropin levels, which correlate with higher rates of gestational hyperthyroidism and nausea and vomiting in pregnancy.^10^ The latter, in conjunction with a history of GERD and cessation of PPI therapy, prompted increased calcium carbonate use causing calcium-alkali syndrome.^11,12^

Only one other reported case made use of renal replacement therapy.^13^ In our patient's case it contributed to rapid and complete recovery of renal function, normalization of calcium levels with an ultimately favorable obstetric outcome.

## Conclusion

This case represents a dramatic presentation of calcium-alkali syndrome despite moderate calcium intake, requiring renal replacement therapy early in twin gestation. Incidence of calcium-alkali syndrome causing severe hypercalcemia is low but is increasing due to widespread availability of calcium-containing antacids.^14^ Fortunately, it is generally associated with good maternal and fetal outcomes if recognized and addressed promptly.
